# Unraveling Obesity: A Five-Year Integrative Review of Transcriptomic Data

**DOI:** 10.3390/ijms262210864

**Published:** 2025-11-09

**Authors:** Svetlana Tarbeeva, Anna Kliuchnikova, Anna Kozlova, Elizaveta Sarygina, Ekaterina Ilgisonis, Elena Ponomarenko

**Affiliations:** Institute of Biomedical Chemistry, 119121 Moscow, Russia

**Keywords:** obesity, transcriptomics, RNA-seq, noncoding RNA, epigenetics, multi-omics

## Abstract

Omics approaches have advanced insight into molecular mechanisms of human obesity. We reviewed transcriptomic studies published between January 2020 and June 2025 that used human tissues or human cell lines and applied high-throughput RNA methods. Across these works three convergent themes emerged: (1) immune–inflammatory activation—particularly interferon-stimulated and innate immune signatures—linked to insulin resistance and visceral adiposity; (2) dysregulation of lipid and energy-metabolism pathways, including reduced lipolysis and β-oxidation in adipose tissue and liver; and (3) epigenetic and post-transcriptional regulation mediated by DNA methylation, histone modification, long noncoding RNAs, microRNAs and circular RNAs. Multi-omics integration (transcriptome with proteome, metabolome and microbiome) improved mechanistic interpretation and biomarker discovery but was limited by cohort heterogeneity and technical variation. We conclude that standardized, integrative multi-omics analyses in well-characterized, longitudinal human cohorts are required to translate molecular signatures into robust biomarkers and personalized therapeutic strategies for obesity.

## 1. Introduction

Obesity is a global epidemic of the 21st century that, according to the World Health Organization, affected one in eight people by 2022: 890 million adults and 160 million children [1]. This epidemic is spreading at an alarming rate—between 1990 and 2022 the combined prevalence of obesity increased in 162 countries (81%) for women and in 140 countries (70%) for men [2]. Obesity is associated with numerous metabolic complications, such as type 2 diabetes mellitus, cardiovascular disease, and certain cancers, making it a comorbidity that contributes to leading causes of mortality. These health burdens also translate into economic costs: experts estimate that in 2019 the expenditures attributable to obesity in eight large high-income countries ranged from 0.8% to 2.4% of gross domestic product [3].

Despite decades of research into the causes and mechanisms of obesity, the molecular processes driving its development remain only partially understood. Obesity is a polygenic, multifactorial disorder in which genetic and environmental factors are intertwined, and phenotypes range from metabolically healthy obesity (MHO) to metabolically unhealthy obesity (MUO). Deep molecular characterization is required to distinguish these phenotypes and to predict associated risks.

One of the key contemporary scientific challenges is to unravel the complex molecular networks that govern metabolic changes in obesity. Omics technologies, including transcriptomic and translatomic approaches, enable the identification of biomarkers and regulatory networks linked to obesity. Transcriptomics (RNA-seq, microarrays, qRT-PCR) provides information on mRNA dynamics, whereas translatomics (ribosome profiling, polysome sequencing) reveals which transcripts are actually translated into proteins and with what efficiency. Combined application of both approaches uncovers layers of post-transcriptional regulation that cannot be resolved by RNA sequencing alone.

Advantages of transcriptomics include high sensitivity to changes in mRNA abundance, the ability to profile individual cell populations (single-cell RNA-seq), and scalability to large sample cohorts. However, RNA-seq does not always correlate with proteomic changes, because translational control and RNA degradation can substantially influence protein levels. Translatome analysis, performed with contemporary methods such as ribosome profiling (Ribo-seq), makes it possible to identify which transcripts are actively translated and how translational output is altered in obesity [4].

Studying the interplay between the transcriptome and the translatome is essential for understanding obesity mechanisms, because many regulatory effects (for example, those induced by endoplasmic reticulum stress or by hypoxia in adipose tissue) are manifested primarily at the level of translation. For instance, a study published in Oxford Academic examined how napping affects circadian rhythms in adipose tissue: individuals who habitually napped showed attenuation of daily oscillations in expression of multiple metabolism- and inflammation-related genes, and the gene *IER3* was found to be expressed 28-fold higher in nappers [5].

Thus, integrative analysis of transcriptomic and translatomic data enables the identification of critical molecular pathways that regulate metabolic dysfunction in obesity and facilitates the discovery of novel targetable markers and therapeutic strategies.

## 2. Results

Some of the key molecules reported in the reviewed studies are summarized in Table 1.

### 2.1. Cellular Models of Adipogenesis

Several recent studies have used diverse cell culture systems to investigate the process of adipocyte formation (adipogenesis) and the related molecular pathways. One of the most informative models is the human Simpson–Golabi–Behmel syndrome (SGBS) preadipocyte cell line, consisting of fibroblast-like cells capable of differentiating into adipocytes. During their conversion into mature adipocytes, large-scale changes in the expression of hundreds of genes and proteins involved in lipid metabolism, mitochondrial function, and gene-regulatory programs have been observed. In particular, stage-specific acetylation sites were identified on fatty acid synthase (FASN) and isocitrate dehydrogenase (IDH) proteins [8], while CD44 (a transmembrane glycoprotein) showed an inverse correlation with adipogenic progression.

Another study using the human SGBS line performed concurrent transcriptomic and proteomic profiling at early and late differentiation stages [22]. Amid hundreds of transcriptomic shifts, the authors demonstrated concordant proteomic changes: classical markers of mature adipocytes increased (for example, lipoprotein lipase *LPL*, fatty acid binding protein 4 *FABP4*, *FASN*, stearoyl-CoA desaturase *SCD*, and apolipoprotein E *APOE*), whereas preadipocyte markers declined. This work produced a reference map of early human adipogenesis that can be used to project results of pharmacological interventions, epigenetic modifications, and studies of noncoding RNAs.

In a BeWo trophoblast model (a human placental cell line), exposure to elevated levels of non-esterified fatty acids (NEFAs), specifically palmitic and oleic acids, induced upregulation of *PLIN2* and *ACSL5*, genes involved in lipid accumulation pathways regulated by the nuclear receptor peroxisome proliferator-activated receptor gamma (PPARγ), a central regulator of adipogenesis [14].

Alongside the above models, the HepG2 cell line (a human hepatocellular carcinoma–derived hepatocyte model) is also widely used to study molecular mechanisms relevant to obesity and related disorders—ranging from lipid accumulation and insulin resistance to pharmacological effects of anti-obesity compounds. Examples include studies of PPARγ ligands [27], investigations of epigenetic regulation of circadian and metabolic pathways [28], and studies of the nutraceutical capsaicin, which exhibits anti-obesity activity [15].

### 2.2. Transcriptomic Studies of Adipose Tissue and Blood

Many recent studies have focused on comparing the transcriptome (the complete set of RNA molecules expressed in a cell or tissue) of adipose tissue in normal-weight individuals and patients with obesity. For example, RNA sequencing (RNA-seq) analysis of subcutaneous and visceral adipose tissue in people with obesity revealed clear sex-dependent differences in the adipose transcriptome [29]. In women versus men, 32 protein-coding genes (e.g., *ROR2* and *SFRP2*) and several long noncoding RNAs (lncRNAs), including XIST and PAX8-AS1, were differentially expressed. Men more frequently exhibited activation of inflammatory pathways and signatures associated with mitochondrial dysfunction. The authors emphasize that accounting for sex differences is critical when interpreting transcriptomic data, because metabolic burden and the development of obesity-related complications can manifest differently in men and women.

These findings are supported by a population-scale study of whole-blood transcriptomes. Analysis of a large cohort (>3000 adults) showed that variability in the whole-blood transcriptome is shaped not only by disease status but also by demographic factors such as age and sex [30]. The authors identified two dominant transcriptomic expression types: an “inflammatory/hematopoietic” profile, which was more common in men, older individuals, and those with overweight/obesity; and an “immune/mitochondrial” profile, which was more typical of women, younger participants, and lean individuals. This study highlights the importance of controlling for age, sex, and population-level factors when interpreting transcriptomic data in clinical cohorts and helps to explain some interstudy heterogeneity in obesity research.

In a separate study of subcutaneous adipose tissue (SAT) from patients with hyperglycemia—measured as elevated glycated hemoglobin (HbA1c), a marker of chronic blood glucose elevation—researchers observed a reduced fraction of mature adipocytes and vascular cells in SAT [31]. At the same time, the activity of several genes linked to restrictions on cell proliferation correlated with pathways of cellular aging (senescence) and telomere maintenance.

Another study used single-cell RNA sequencing (scRNA-seq) to profile stromal and immune cells in SAT from people living with human immunodeficiency virus (HIV) [9]. In patients with increased visceral adipose tissue (VAT), there was an expansion of intermediate macrophages and of fibroblasts expressing the *MYOC* gene (encoding the extracellular matrix protein myocilin). These changes were associated with elevated expression of extracellular matrix and inflammatory genes and with decreased activity of lipolytic pathways. This cellular and molecular pattern is consistent with progressive adipose tissue fibrosis and chronic inflammation, hallmark features of metabolic dysregulation in obesity.

A recent meta-analysis of publicly available blood transcriptome datasets compared metabolically unhealthy obesity (MUO) with metabolically healthy obesity (MHO). The pooled analysis identified a robust signature of approximately 190 differentially expressed genes enriched for innate and adaptive immune response pathways, thrombo-inflammatory signaling, and cell-adhesion processes. Network analysis revealed MUO “hub” genes (network central nodes), including *EGF*, *STAT3*, *IL1B*, *PF4*, *SELP*, and *ITGA2B*. Practically, these findings provide a basis for stratifying people with obesity by cardiometabolic risk and for developing blood-based transcriptomic biomarker panels suitable for clinical screening [32].

### 2.3. Obesity and Comorbid Conditions

As noted above, obesity is a complex systemic condition that most often presents clinically in the context of comorbid diseases rather than as an isolated primary complaint. Consequently, many studies have examined how obesity modifies the molecular signatures of comorbid conditions.

Obesity is most frequently studied in conjunction with type 2 diabetes mellitus (T2DM). Integrated proteomic and transcriptomic analysis of liver tissue from patients with obesity and T2DM identified *CMPK1* as significantly elevated compared with obese patients without diabetes [7]. This finding highlights *CMPK1* as a candidate research biomarker that may help to stratify obese individuals by hepatic metabolic phenotype, but it requires further validation before any diagnostic application can be considered. In another study of patients undergoing bariatric surgery, transcriptional differences were found between individuals whose diabetes remitted after surgery and those who did not; genes such as *STK4* (also written as *STK4* or *SKT4*), *SIRT1*, and members of the tumor necrosis factor (TNF) family were among the predictors of remission. These transcriptional signatures could form the basis of prognostic models for surgical response [11].

In metabolic dysfunction-associated steatotic liver disease (MASLD; formerly NAFLD), which often coexists with obesity, investigators observed pronounced dysregulation of autophagy—the cellular process that degrades damaged organelles and proteins [10]. Lysosomal proteases (cathepsins) were upregulated, and cathepsin D (*CTSD*) was increased at the mRNA, protein, and serum levels, suggesting *CTSD* as a candidate biomarker of advanced MASLD.

Cross-disease molecular mechanisms shared between obesity and other metabolic disorders have also been revealed by in silico analyses of previously published data. For example, bioinformatic comparison of transcriptomes in polycystic ovary syndrome (PCOS), obesity, and T2DM identified jointly dysregulated genes (including *IGF2R*, the insulin-like growth factor 2 receptor) and network “hub” proteins such as *STAT3*, *IL1B*, and *PF4* [19]. These results point to overlapping pathogenic mechanisms of inflammation and growth regulation across these conditions. In a related observation, whole-blood transcriptomics demonstrated that individuals with insulin resistance within obesity exhibit excessive expression of immune response genes compared with metabolically healthy obese subjects, underscoring the role of systemic inflammation in shaping the clinical phenotype of obesity [33].

Obesity also modifies the tumor microenvironment and tumor transcriptomes. For instance, in early-stage non-small-cell lung cancer (NSCLC), statin use was associated with improved recurrence-free survival primarily in overweight and obese patients; in these patients, statin therapy correlated with increased tumor expression of cytotoxic immune markers such as granzymes and interferon-γ (IFN-γ), suggesting augmented antitumor immunity in the setting of obesity [34]. In visceral adipocytes of colorectal cancer patients—particularly when combined with obesity—pathways related to extracellular matrix remodeling, cell adhesion, and transforming growth factor-β (TGF-β) signaling were activated, whereas obesity alone tended to be associated with proinflammatory cascades [35].

In summary, transcriptomic studies demonstrate that obesity alters molecular signatures across a broad spectrum of diseases—from metabolic disorders to malignancies—and that these changes can serve both as diagnostic/prognostic markers and as targets for novel therapeutic approaches.

### 2.4. Epigenetic and Noncoding Regulators

Numerous studies demonstrate that alterations in DNA methylation are closely associated with the development of obesity. A 2023 analysis of publicly available genome-wide data identified 107 DNA methylation sites (CpG sites) that are associated with levels of 84 metabolites, predominantly lipids [17]. Integration of methylation data with gene expression profiles revealed putative mediators of these relationships—genes such as *ILRUN*, *POC5*, *FDFT1*, *NEIL2*, and others—whose methylation changes may affect metabolic pathways and contribute to obesity pathogenesis.

In another integrative methylome–transcriptome analysis, seven key genes were identified—*CCRL2*, *GPT*, *LGALS12*, *PC*, *SLC27A2*, *SLC4A4*, and *TTC36*—that were hypermethylated in obesity and concurrently showed reduced expression [20]. In addition, subcutaneous adipose tissue (SAT) from individuals with obesity displayed shifts in immune cell composition: an increased proportion of M0 macrophages (a non-activated, “naïve” macrophage population) and a decreased fraction of T follicular helper (Tfh) cells. The abundance of these cellular subpopulations correlated with the expression of the genes listed above.

Beyond DNA methylation, noncoding RNAs play a significant regulatory role in adipogenesis. For example, the adipocyte-enriched long noncoding RNA (lncRNA) lncRAP2 forms a complex with the RNA-binding protein IGF2BP2 (insulin-like growth factor 2 mRNA-binding protein 2), stabilizing mRNAs of adipogenic regulators, including the adiponectin transcript [21]. Studies in the murine 3T3-L1 cell line, in primary adipocytes, and in adipose tissue showed that suppression of lncRAP2 or IGF2BP2 reduces lipolysis in adipocytes. Moreover, both factors are downregulated in adipose tissue from people with obesity and type 2 diabetes. The lncRAP2–IGF2BP2 complex promotes adipose tissue development, and genetic variants in these elements are associated with susceptibility to obesity-linked diabetes.

Analyses of noncoding RNA expression profiles revealed widespread rewiring of regulatory networks in obesity. For instance, in patients with obesity and type 2 diabetes (O-T2DM), blood profiling detected 442 differentially expressed circular RNAs (circRNAs) and 2756 differentially expressed messenger RNAs (mRNAs) [26]. Functional annotation of these sets implicated immune responses, oxidative phosphorylation, and apoptosis-regulating pathways. Network analysis highlighted the circRNA hsa_circ_0060614, which is upregulated in O-T2DM and functions as a “sponge” for microRNA miR-4668-3p, thereby regulating the mRNA of MT2A (the gene encoding metallothionein-2A, a protein involved in metal ion binding and protection against oxidative stress).

Another notable noncoding regulator is the circular RNA circMAPK9, whose level is increased in visceral adipose tissue of patients with obesity [13]. circMAPK9 also acts as a competing endogenous RNA (a “RNA sponge”) for microRNA miR-1322, sequestering it and relieving its repressive effect on the *FTO* gene (a gene implicated in body weight regulation). Reduced miR-1322 activity results in upregulation of *FTO*, which in turn promotes fat accumulation. In a separate study of omental adipose tissue, 11 key lncRNAs and 4 circRNAs implicated in lipid metabolism and inflammation were identified in obesity [25].

Significant alterations in circulating nucleic acids have also been reported in pediatric obesity: four microRNAs (miR-328-3p, miR-1301-3p, miR-4685-3p, miR-6803-3p) were significantly downregulated and correlated with measures of adiposity and with serum leptin (the hormone that regulates appetite and energy balance). These microRNAs target a large set of mRNAs, and functional analyses indicate their involvement in key signaling cascades, including the PI3K–Akt pathway (phosphoinositide 3-kinase–Akt pathway), which centrally regulates glucose metabolism, cell growth, and survival [36].

### 2.5. Multi-Omics Approaches

Contemporary studies increasingly combine transcriptomic data with other omics layers to obtain a deeper understanding of the molecular mechanisms of obesity. For example, integration of transcriptomic and proteomic data during early adipogenesis of adipose-derived stem cells showed that out of approximately 3400 measured proteins, 33 underwent significant changes as early as 24 h after induction of differentiation. Among these were FADS1, FADS2, and LAMB1, changes that were concordant with transcriptomic shifts [37].

In children with obesity, an integrated blood multi-omics analysis combining transcriptomics and metabolomics identified 599 differentially expressed genes (25 of which were immune-related, e.g., genes involved in neutrophil degranulation and interferon pathways) and 71 metabolites that differed from controls. Notably, lauric acid was strongly correlated with body mass index and was included in a panel of 14 molecular markers (genes and metabolites) selected by a Random Forest model that discriminated obese from non-obese children with high accuracy [24]. The same study also examined the stool microbiome and reported a significant increase in the abundance of Firmicutes in obese children.

In a pilot metatranscriptomic study of duodenal mucosa from individuals with severe obesity versus lean controls, both host transcripts and microbial activity were profiled simultaneously [38]. The authors found altered expression of about 40 human genes in the duodenal tissue of individuals with severe obesity, affecting carbohydrate, lipid, and protein metabolism, epithelial differentiation, and immune pathways. Concurrently, they observed restructuring of the microbial metatranscriptome (approximately 55 differentially expressed microbial transcripts) and shifts in activity among major microbial taxa (changes in activity of members of Firmicutes and Proteobacteria). These results indicate coordinated alterations in the functional activity of the upper gastrointestinal tract and its associated microbiota in severe obesity.

A transcriptome–metabolome analysis of blood from breast cancer patients with obesity revealed more than 180 differentially expressed genes and nearly 100 metabolites; notably, *OLFM4* expression was markedly elevated in the obese subgroup [23]. This finding may reflect immune dysregulation associated with cancer in the context of excess adiposity.

Particularly important for understanding obesity pathogenesis is the integration of transcriptomic and translatomic data. In the only study that met our inclusion criteria and combined RNA-seq with ribosome profiling (Ribo-seq), supplementation with betaine markedly reduced hepatic triglyceride accumulation in mice fed a high-fat diet and simultaneously modulated expression of lipid genes in HepG2 cells [39]. Importantly, betaine’s effects were more pronounced at the translational level: the translational efficiency of key lipogenic enzymes (*IDI1*, *CYP51A1*, *TM7SF2*, *APOA4*) was decreased, whereas transcript-level changes were less significant.

## 3. Discussion

Analysis of the recent literature revealed several methodological features that must be taken into account when interpreting transcriptomic and translatomic data. During the article screening stage, it became apparent that a substantial proportion of publications are devoted not to clinical cohorts of people with obesity but to cellular models of adipogenesis or to animal experiments. This bias is explained in part by the technical and ethical difficulties of obtaining human adipose tissue biopsies and by the high interindividual variability of adipose tissue, which complicates the assembly of homogeneous patient cohorts. Many articles indexed under the tag “obesity” therefore reflect fundamental studies of the molecular mechanisms of adipocyte differentiation rather than direct clinical investigation of human obesity. Even among clinical studies, patient heterogeneity remains a major challenge. Differences in sex, age, comorbid conditions (for example, type 2 diabetes mellitus, metabolic dysfunction-associated steatotic liver disease, human immunodeficiency virus infection, and cancer), and fat distribution (visceral versus subcutaneous adiposity) make it difficult to construct representative control groups. This complexity largely explains the diversity of transcriptomic signatures reported across different studies.

It is also noteworthy that a large number of publications focus on the protein FTO (fat mass and obesity-associated protein), which functions as an m6A RNA demethylase. Because of its role in post-transcriptional regulation and adipogenesis, FTO has become one of the most frequently studied targets in genetic manipulation models of obesity.

By contrast, investigations of the translatome in obesity remain severely limited. Only two studies identified by our search employed ribosome profiling (Ribo-seq), and of these only one met our inclusion criteria for detailed analysis. This highlights that translatome research in the context of obesity is largely an uncharted area and represents a promising direction for future work.

Despite these limitations, an extensive body of transcriptomic and multi-omic data on obesity has accumulated in recent years. Synthesis of the literature indicates that coordinated changes in metabolic and immune processes lie at the core of obesity pathogenesis. Numerous studies confirm that obesity is associated with systemic inflammation: interferon-stimulated genes are upregulated in the blood of insulin-resistant subjects [33], and extracellular matrix and proinflammatory genes are increased in adipose tissue in the setting of visceral adiposity [34]. At the same time, transcription of genes involved in lipolysis and fatty-acid β-oxidation is often suppressed in adipose depots and in the liver, particularly when obesity coexists with type 2 diabetes mellitus (T2DM) or metabolic dysfunction-associated steatotic liver disease (MASLD), further aggravating metabolic dysfunction.

Modern omics technologies have underscored the importance of epigenetic regulation and noncoding RNAs. Global DNA methylation and histone modifications modulate expression of metabolic genes; for example, high-fat diet (HFD)-induced epigenetic shifts can “unlock” lipogenic and MAPK signaling genes [40]. Noncoding RNAs (long noncoding RNAs, microRNAs, circular RNAs) act as key regulators of adipogenesis and metabolic pathways: the adipocyte lncRNA lncRAP2 stabilizes adipocyte transcripts, while circMAPK9 and other noncoding RNAs modulate signaling axes such as FTO and MT2A in obesity-associated T2DM. These findings indicate that transcriptional profiles in obesity are shaped across multiple regulatory layers.

Another important class of obesity regulators consists of G protein-coupled receptor (GPCR) pathways and their ligands. GPCRs sense hormones and metabolites and govern appetite and energy balance [41]. For example, GLP-1 and GIP receptor agonists, which signal through GPCRs, are emerging therapies for weight loss. Although our transcriptomic search did not retrieve studies focusing on these pathways, many anti-obesity drugs target GPCRs and peptide hormones (e.g., GLP-1R/GIPR dual agonists).

Interdisciplinary and multi-omics approaches are also crucial. Combining RNA sequencing with proteomics, metabolomics, and microbiome profiling permits construction of an integrated disease view. For instance, a multi-omics blood study in children linked transcriptomic, metabolomic, and microbiome alterations and illustrated how immunometabolites (for example, lauric acid) interact with the microbial ecosystem in pediatric obesity [24].

Nevertheless, the accumulated data also highlight substantial challenges. High interindividual and tissue heterogeneity complicates the interpretation of expression profiles. Sex differences in adipose transcriptomes [29] and the influence of comorbid conditions (HIV, cancer, diabetes, etc.) require stratified analyses. From a technical standpoint, cellular composition must be accounted for: blood transcriptome analyses should be adjusted for leukocyte proportions, as performed by the authors of [33].

Overall, synthesis of recent findings points to several key themes. First, obesity is tightly linked to chronic inflammation and impaired immune surveillance: combinations of immune markers (interferons, granzymes, chemokines) together with metabolic pathways (lipogenesis, fatty-acid oxidation) form a reproducible “transcriptomic signature” of obesity and its comorbidities. Second, immunometabolic pathways represent promising therapeutic targets; however, interventions must be precise. For example, targeted modulation of NF-κB signaling may reduce adipose inflammation but could induce adipocyte apoptosis if applied indiscriminately [42]. Agents that affect inflammatory and fibrotic cascades as well as epigenetic regulators (for example, inhibitors of FTO) are under investigation as potential therapeutics.

Finally, “big data” and bioinformatics remain pivotal. Advances in machine learning and integrative analysis of omics datasets will help to extract disease patterns and identify novel biomarkers, as illustrated by a Random Forest model for pediatric obesity [24].

We note that some molecular signals identified in integrative studies (for example, *CMPK1* in liver tissue [7]) are promising as research or prognostic biomarkers rather than as immediate clinical diagnostics. Measurement of *CMPK1* currently relies on proteomic workflows or IHC, which are not practical replacements for routine glycemic assays.

However, standardization of protocols and analytical frameworks is essential to ensure comparability across studies. Future research should prioritize prospective and longitudinal cohort designs (for example, pre- and post-intervention sampling) and should combine multiple interventions (diet, pharmacotherapy, exercise) with omics profiling. In this way, transcriptomic research on obesity in recent years has deepened our understanding of the molecular basis of the disease and opened new avenues for personalized diagnosis and therapy.

## 4. Materials and Methods

A systematic literature search was performed in PubMed covering the last five years (January 2020–June 2025). The main inclusion criteria were: (1) presence of experimental data obtained on human tissue samples or human cell lines; (2) use of transcriptomic (RNA-seq, microarray) or translatomic methods (Ribo-seq, polysome profiling); and (3) a clear link between the study and the phenomenon of obesity or its metabolic complications. Excluded were animal studies, preprints, reviews without primary data, and publications with poor methodological quality.

The primary PubMed query was constructed as follows: (obesity[Title/Abstract]) AND (transcriptome[Title/Abstract] OR translatome[Title/Abstract]) NOT animals[mh] NOT review[pt] NOT comment[pt] NOT case reports[pt].

The screening strategy intentionally targeted publications explicitly annotated with transcriptome/translatome-related keywords to ensure focus and consistency across included studies. Because indexing practices and keyword choice vary by discipline, papers whose primary emphasis was on alternative molecular routes—e.g., receptor pharmacology, peptide-hormone signalling, post-translational regulators, or metabolic enzyme pathways—and which are frequently tagged under different MeSH terms or keyword sets, may not have been captured by our query and therefore could be underrepresented in this review.

Out of 158 initially retrieved records, screening for relevance yielded 36 studies that met the criteria and which used patient samples or human cell lines. From each article we extracted the key experimental methods (e.g., next-generation sequencing RNA-seq, RT-qPCR, proteomic analysis) and the principal results. The collated material and a brief description of each publication are provided in Supplementary Table S1.

## 5. Conclusions

Human transcriptomic research in obesity demonstrates that obesity is a complex metabolic omics phenomenon. Key findings include the identification of genes and proteins altered in obesity and its comorbidities, and the delineation of recurrent regulatory signatures involving inflammation, lipid metabolism, and cell-cycle control. These results support the concept of chronic inflammation and disrupted metabolic homeostasis in obesity. At the same time, they emphasize the need for further research: integrative multi-omics studies and long-term clinical cohorts are required to link dynamic gene expression changes with clinical outcomes. In the future, combining transcriptomics, translatomics, epigenomics, and proteomics across tissues (adipose, liver, muscle, gut, etc.) will provide a deeper mechanistic understanding of obesity pathogenesis. This expanded molecular insight can ultimately lead to novel targeted therapies and to personalization of treatment for obesity and its associated diseases.

## Figures and Tables

**Table 1:** Key molecules associated with obesity based on transcriptomic studies (↑, upregulation/activation; ↓, downregulation/inhibition of genes or pathways).

Gene/Molecule	Experimental Methods	Study Group	Biological Context	Ref.
*EYA4*, *MBOAT2*, *SCGB2A1* (genes)	RNA-seq (TCGA), bioinformatics analysis	Tumor tissue of obese endometrial cancer patients	Immune-related prognostic signature in obesity-associated endometrial cancer	[6]
*CMPK1* (gene)	Proteomics (TMT LC-MS/MS), transcriptomics (GEO), IHC	Liver tissue from obese patients with vs. without T2DM	*CMPK1* upregulated in T2DM vs. obese controls; potential research/prognostic biomarker	[7]
*IER3* (gene), *VDAC2P2* (pseudogene)	24 h adipose explant RNA-seq	Subcutaneous fat from nappers vs. non-nappers (with abdominal obesity)	Blunted circadian rhythms in nappers (*IER3* ↑ in nappers); altered metabolic/inflammatory genes	[5]
CD44 (protein), FASN_K673, IDH2_K272 (acetylation sites)	RNA-seq, acetylomics, phosphoproteomics (LC-MS/MS)	SGBS (human) and 3T3-L1 (mouse) preadipocytes differentiating	Stage-specific protein acetylation in adipogenesis (metabolic/insulin pathways)	[8]
*MYOC* (gene)	Single-cell and bulk RNA-seq, CT imaging	Subcutaneous fat of HIV^+^ patients (high vs. low visceral fat)	Expansion of profibrotic MYOC^+^ fibroblasts with more visceral fat (↑ ECM/inflammation, ↓ lipolysis genes)	[9]
*CTSD* (gene)	RNA-seq, ELISA (serum), Western blot	Liver and visceral fat from obese patients (severe vs. mild MASLD)	Autophagy/lysosome pathway dysregulated; cathepsin D (*CTSD*) expression increases with MASLD severity	[10]
*SKT4* (gene), *SIRT1* (gene), TNF family genes	Whole-transcriptome microarray, RT-qPCR	PBMCs from obese T2DM patients (remitters vs. non-remitters post-surgery)	Differentially expressed Hippo and Sirtuin pathway genes predict diabetes remission	[11]
*HIF1A*, *NOX1*, *NOX2*, *IL1B*, *IL8*, *IL10*, *VEGFA* (genes)	RNA-seq, pulse wave velocity (PWV), RT-qPCR, WB	Obese adults with vitamin D deficiency vs. controls	Inflammation and oxidative stress genes upregulated in vitamin D–deficient obesity	[12]
circMAPK9 (circRNA), *FTO* (gene)	circRNA-seq, RT-qPCR, FISH, luciferase assays	Visceral adipose from obese vs. lean subjects	circMAPK9 promotes adipogenesis via sponging miR-1322 and upregulating *FTO*	[13]
*ACSL5* (gene), *PLIN2* (gene), FADS2 (enzyme)	Lipidomics (GC-MS, TLC), LC-MS/MS metabolomics, transcriptomics	Human trophoblast (BeWo cells) treated with palmitate or oleate	Saturated/unsaturated NEFAs alter trophoblast lipid metabolism; *ACSL5* and *PLIN2* are upregulated	[14]
*CTH*, *VEGFA*, *PCK2*, *IGFBP3* (genes)	HepG2 culture glucose uptake assay, RNA-seq, KEGG analysis, RT-qPCR	Human HepG2 hepatocytes + capsaicin	Capsaicin enhances glucose uptake in hepatocytes; upregulates genes controlling metabolism (*CTH*, *VEGFA*, *PCK2*, *IGFBP3*)	[15]
*CLU* (clusterin gene), Collagen (ECM protein)	Adipose tissue RNA-seq, serum ELISA (clusterin)	Abdominal SAT/VAT from lean vs. obese subjects	VAT clusterin levels correlate with adipose insulin resistance and collagen accumulation	[16]
*ILRUN*, *POC5*, *FDFT1*, *NEIL2* (genes)	GWAS analysis, Mendelian randomization of DNA methylation	In silico blood methylation-metabolome datasets	CpG methylation sites in these genes regulate metabolite levels; connected to obesity/diabetes pathways	[17]
CNDP2, HSPA9, GANAB, ATP5B (proteins)	Electrical pulse stimulation of myotubes; EV proteomics (LC-MS/MS); miRNA profiling	Exosomes/MVs from skeletal muscle of obese T2D donors ± “exercise” mimic	Exercise-mimetic changes EV cargo; notable increase in CNDP2 (Lac-Phe pathway enzyme) and its regulatory miRNA	[18]
*IGF2R* (gene)	Transcriptome meta-analysis (PPI network, docking)	In silico comparison of PCOS with obesity, T2D, CVD datasets	Shared molecular mechanisms in PCOS and metabolic diseases; *IGF2R* (IGF2 receptor) is a key hub	[19]
*CCRL2*, *GPT*, *LGALS12*, *PC*, *SLC27A2*, *SLC4A4*, *TTC36* (genes)	DNA methylation (450 K array), RNA-seq (GEO data), immune deconvolution	SAT from obese vs. lean individuals	Seven obesity-related genes identified (methylation-regulated); linked to altered immune cell composition in fat	[20]
*lncRAP2* (lncRNA), IGF2BP2 (protein)	RNA interactome (RAP-MS), quantitative proteomics, ribosome profiling, FISH	Mouse 3T3-L1 and human adipocytes (plus genetic cohorts)	Conserved adipocyte lncRAP2–IGF2BP2 complex stabilizes adipogenic mRNAs (e.g., adiponectin), enhancing adipogenesis	[21]
*ADIPOQ*, *LPL*, *FABP4*, *FASN*, *SCD*, *APOE* (genes)	Shotgun proteomics (LC-MS/MS), RNA-seq	Human SGBS cells: preadipocytes vs. 24 h adipocytes	Classic adipocyte differentiation markers (adiponectin, *LPL*, *FABP4*, *FASN*, *SCD*, ApoE) strongly upregulated	[22]
*OLFM4* (gene)	Whole-blood RNA-seq, serum metabolomics	Breast cancer patients: obese (BMI ≥ 30) vs. non-obese	Obesity alters blood transcriptome/metabolome in breast cancer; *OLFM4* notably overexpressed in obese BC patients	[23]
Lauric acid (metabolite), *OLFM4*, *CRISP3* (genes)	Blood RNA-seq, serum metabolomics, fecal 16S rRNA sequencing	Children with simple obesity vs. lean controls	Multi-omics obesity biomarkers: lauric acid strongly correlates with BMI (AUC = 0.82); immune genes (e.g., *OLFM4*, *CRISP3*) highlighted	[24]
ENST00000605862, ENST00000558885, ENST00000686149 (lncRNAs)	Whole-transcriptome RNA-seq (ribo-depletion), qRT-PCR validation, network analysis	Omental adipose from obese vs. control adults	Dysregulated noncoding RNAs in obesity; these lncRNAs are key regulatory nodes in metabolic pathways	[25]
hsa_circ_0060614 (circRNA)	Blood RNA-seq (circRNA + mRNA), WGCNA, pathway analysis	Obesity-associated T2DM (O-T2DM) vs. healthy controls	circRNA–miRNA–mRNA regulatory network in O-T2DM: hsa_circ_0060614 upregulated, sponges miR-4668-3p to regulate MT2A	[26]

## Data Availability

No new data were created or analyzed in this study. Data sharing is not applicable to this article.

## Supplementary Materials

The following supporting information can be downloaded at: https://www.mdpi.com/article/10.3390/ijms262210864/s1.

## Abbreviations

The following abbreviations are used in this manuscript:

BMI	Body Mass Index
circRNA	Circular RNA
ECM	Extracellular Matrix
ELISA	Enzyme-Linked Immunosorbent Assay
EV	Extracellular Vesicle
FISH	Fluorescence In situ Hybridization
GC-MS	Gas Chromatography–Mass Spectrometry
GEO	Gene Expression Omnibus
GWAS	Genome-Wide Association Study
HbA1c	Glycated Hemoglobin
HFD	High-Fat Diet
IFN-γ	Interferon Gamma
Ig	Immunoglobulin
IHC	Immunohistochemistry
KEGG	Kyoto Encyclopedia of Genes and Genomes
LC-MS/MS	Liquid Chromatography with Tandem Mass Spectrometry
lncRNA	Long Noncoding RNA
MAPK	Mitogen-Activated Protein Kinase
MASLD	Metabolic Dysfunction-Associated Steatotic Liver Disease
MHO	Metabolically Healthy Obesity
miRNA/miR	MicroRNA
mRNA	Messenger RNA
MUO	Metabolically Unhealthy Obesity
MV	Microvesicles
NAFLD	Non-Alcoholic Fatty Liver Disease
NEFA	Non-Esterified Fatty Acid
NSCLC	Non-Small-Cell Lung Cancer
PBMCs	Peripheral Blood Mononuclear Cells
PCOS	Polycystic Ovary Syndrome
PPARγ	Peroxisome Proliferator-Activated Receptor Gamma
PPI	Protein–Protein Interaction
PWV	Pulse Wave Velocity
qRT-PCR/RT-qPCR	Quantitative Real-Time Polymerase Chain Reaction
Ribo-seq	Ribosome Profiling Sequencing
SAT	Subcutaneous Adipose Tissue
scRNA-seq	Single-Cell RNA Sequencing
T2DM	Type 2 Diabetes Mellitus
TCGA	The Cancer Genome Atlas
TGF-β	Transforming Growth Factor Beta
TLC	Thin-Layer Chromatography
TMT	Tandem Mass Tag
TNF	Tumor Necrosis Factor
VAT	Visceral Adipose Tissue
WB	Western Blot
WGCNA	Weighted Gene Co-expression Network Analysis

## References

[1] Obesity and Overweight. https://www.who.int/news-room/fact-sheets/detail/obesity-and-overweight.

[2] (2024). NCD Risk Factor Collaboration Worldwide trends in underweight and obesity from 1990 to 2022: A pooled analysis of 3663 population-representative studies with 222 million children, adolescents, and adults. Lancet.

[3] Okunogbe A., Nugent R., Spencer G., Ralston J., Wilding J. (2021). Economic impacts of overweight and obesity: Current and future estimates for eight countries. BMJ Glob. Health.

[4] Kozlova A., Sarygina E., Ilgisonis E., Tarbeeva S., Ponomarenko E. (2024). The Translatome Map: RNC-Seq vs. Ribo-Seq for Profiling of HBE, A549, and MCF-7 Cell Lines. Int. J. Mol. Sci..

[5] Rodríguez-Martín M., Pérez-Sanz F., Zambrano C., Luján J., Ryden M., Scheer F.A.J.L., Garaulet M. (2024). Circadian transcriptome oscillations in human adipose tissue depend on napping status and link to metabolic and inflammatory pathways. Sleep.

[6] Tong Y., Zhu T., Xu F., Yang W., Wang Y., Zhang X., Chen X., Liu L. (2024). Construction of an immune-related gene prognostic model for obese endometrial cancer patients based on bioinformatics analysis. Heliyon.

[7] Zhao K., Mao R., Yi W., Ren Z., Liu Y., Yang H., Wang S., Feng Z. (2024). Integrated transcriptomics and proteomics identified CMPK1 as a potential biomarker for type 2 diabetes mellitus. Diabetes Metab. Syndr. Obes..

[8] Aldehoff A.S., Karkossa I., Goerdeler C., Krieg L., Schor J., Engelmann B., Wabitsch M., Landgraf K., Hackermüller J., Körner A. (2024). Unveiling the dynamics of acetylation and phosphorylation in SGBS and 3T3-L1 adipogenesis. iScience.

[9] Bailin S.S., Gabriel C.L., Gangula R.D., Hannah L., Nair S., Carr J.J., Terry J.G., Silver H.J., Simmons J.D., Mashayekhi M. (2024). Single-Cell Analysis of Subcutaneous Fat Reveals Profibrotic Cells That Correlate with Visceral Adiposity in HIV. J. Clin. Endocrinol. Metab..

[10] Meroni M., De Caro E., Chiappori F., Longo M., Paolini E., Mosca E., Merelli I., Lombardi R., Badiali S., Maggioni M. (2024). Hepatic and adipose tissue transcriptome analysis highlights a commonly deregulated autophagic pathway in severe MASLD. Obesity.

[11] Rashid M., Al Qarni A., Al Mahri S., Mohammad S., Khan A., Abdullah M.L., Lehe C., Al Amoudi R., Aldibasi O., Bouchama A. (2023). Transcriptome changes and metabolic outcomes after bariatric surgery in adults with obesity and type 2 diabetes. J. Endocr. Soc..

[12] Elmoselhi A.B., Bouzid A., Allah M.S., Ibrahim Z., Bajbouj K., Abou Assaleh R.S., Venkatachalam T., Madkour M., Siddiqui R., Khan N.A. (2023). Unveiling the molecular Culprit of arterial stiffness in vitamin D deficiency and obesity: Potential for novel therapeutic targets. Heliyon.

[13] Chen S., Song P., Wang Y., Wang Z., Xue J., Jiang Y., Zhou Y., Zhao J., Tang L. (2023). CircMAPK9 promotes adipogenesis through modulating hsa-miR-1322/FTO axis in obesity. iScience.

[14] Easton Z.J.W., Sarr O., Zhao L., Buzatto A.Z., Luo X., Zhao S., Li L., Regnault T.R.H. (2023). An Integrated Multi-OMICS Approach Highlights Elevated Non-Esterified Fatty Acids Impact BeWo Trophoblast Metabolism and Lipid Processing. Metabolites.

[15] Zeng H., Shi N., Peng W., Yang Q., Ren J., Yang H., Chen L., Chen Y., Guo J. (2023). Effects of capsaicin on glucose uptake and consumption in hepatocytes. Molecules.

[16] Wang Y., Yu H., Ma X., Wang Y., Liu W., Zhang H., Chen W., Yu S., Bao Y., Yang Y. (2023). Clusterin is closely associated with adipose tissue insulin resistance. Diabetes Metab. Res. Rev..

[17] Nikpay M. (2023). Genome-wide search identified DNA methylation sites that regulate the metabolome. Front. Genet..

[18] Aas V., Øvstebø R., Brusletto B.S., Aspelin T., Trøseid A.-M.S., Qureshi S., Eid D.S.O., Olstad O.K., Nyman T.A., Haug K.B.F. (2023). Distinct microRNA and protein profiles of extracellular vesicles secreted from myotubes from morbidly obese donors with type 2 diabetes in response to electrical pulse stimulation. Front. Physiol..

[19] Hossain M.A., Al Ashik S.A., Mahin M.R., Al Amin M., Rahman M.H., Khan M.A., Emran A.A. (2022). Systems biology and in silico-based analysis of PCOS revealed the risk of metabolic disorders. Heliyon.

[20] Wu P., Guo L., Li X., Du Y., Lin X., Ma X., Lin Y., Wen C., Yang C., Liu N. (2022). Comprehensive analysis of epigenomics and transcriptome data to identify potential target genes associated with obesity. Front. Genet..

[21] Alvarez-Dominguez J.R., Winther S., Hansen J.B., Lodish H.F., Knoll M. (2022). An adipose lncRAP2-Igf2bp2 complex enhances adipogenesis and energy expenditure by stabilizing target mRNAs. iScience.

[22] Kalkhof S., Büttner P., Krieg L., Wabitsch M., Küntzel C., Friebe D., Landgraf K., Hanschkow M., Schubert K., Kiess W. (2020). In Depth Quantitative Proteomic and Transcriptomic Characterization of Human Adipocyte Differentiation using the SGBS Cell Line. Proteomics.

[23] Hassan M.A., Al-Sakkaf K., Shait Mohammed M.R., Dallol A., Al-Maghrabi J., Aldahlawi A., Ashoor S., Maamra M., Ragoussis J., Wu W. (2020). Integration of transcriptome and metabolome provides unique insights to pathways associated with obese breast cancer patients. Front. Oncol..

[24] Ren Y., Huang P., Zhang L., Tang Y., He S., Li H., Huang X., Ding Y., Liu L., Liu L. (2024). Multi-omics landscape of childhood simple obesity: Novel insights into pathogenesis and biomarkers discovery. Cell Biosci..

[25] Zhang Y., Chen A., Lu S., Liu D., Xuan X., Lei X., Zhong M., Gao F. (2025). Noncoding RNA profiling in omentum adipose tissue from obese patients and the identification of novel metabolic biomarkers. Front. Genet..

[26] Zhen X.-J., Hu R.-T., Liu N.-N., Dou J.-F., Wu T., Zhang Y.-L., Zhang C.-Y., Ma L., Jiang G.-J. (2025). CircRNA-mediated ceRNA regulatory networks: Transcriptomic insights into obesity type 2 diabetes progression and treatment strategies. Diabetol. Metab. Syndr..

[27] Cheng V., Reddam A., Bhatia A., Hur M., Kirkwood J.S., Volz D.C. (2021). Utilizing systems biology to reveal cellular responses to peroxisome proliferator-activated receptor γ ligand exposure. Curr. Res. Toxicol..

[28] Carbone A., De Santis E., Cela O., Giambra V., Miele L., Marrone G., Grieco A., Buschbeck M., Capitanio N., Mazza T. (2021). The histone variant macroh2a1 impacts circadian gene expression and cell phenotype in an in vitro model of hepatocellular carcinoma. Biomedicines.

[29] Rey F., Messa L., Pandini C., Maghraby E., Barzaghini B., Garofalo M., Micheletto G., Raimondi M.T., Bertoli S., Cereda C. (2021). RNA-seq Characterization of Sex-Differences in Adipose Tissue of Obesity Affected Patients: Computational Analysis of Differentially Expressed Coding and Non-Coding RNAs. J. Pers. Med..

[30] Schmidt M., Hopp L., Arakelyan A., Kirsten H., Engel C., Wirkner K., Krohn K., Burkhardt R., Thiery J., Loeffler M. (2020). The human blood transcriptome in a large population cohort and its relation to aging and health. Front. Big Data.

[31] Paulí S., Oliveras-Cañellas N., Moreno-Navarrete J.M., Castells-Nobau A., Ortega F.J., Rodriguez-Hermosa J.I., Castro E., Zhang B., Zhou Y., Gómez-Ambrosi J. (2025). Cellular composition and transcriptomics of subcutaneous adipose tissue linked to blood glycated haemoglobin. Eur. J. Clin. Investig..

[32] Wang Q., Wang S., Zhuang Z., Wu X., Gao H., Zhang T., Zou G., Ge X., Liu Y. (2025). Identification of potential crucial genes and mechanisms associated with metabolically unhealthy obesity based on the gene expression profile. Front. Genet..

[33] Kalafati M., Kutmon M., Evelo C.T., van der Kallen C.J.H., Schalkwijk C.G., Stehouwer C.D.A., Consortium B.I.O.S., Blaak E.E., van Greevenbroek M.M.J., Adriaens M. (2021). An interferon-related signature characterizes the whole blood transcriptome profile of insulin-resistant individuals-the CODAM study. Genes Nutr..

[34] Patnaik S.K., Petrucci C., Barbi J., Seager R.J., Pabla S., Yendamuri S. (2021). Obesity-Specific Association of Statin Use and Reduced Risk of Recurrence of Early Stage NSCLC. JTO Clin. Res. Rep..

[35] Tait S., Baldassarre A., Masotti A., Calura E., Martini P., Varì R., Scazzocchio B., Gessani S., Del Cornò M. (2020). Integrated Transcriptome Analysis of Human Visceral Adipocytes Unravels Dysregulated microRNA-Long Non-coding RNA-mRNA Networks in Obesity and Colorectal Cancer. Front. Oncol..

[36] Kim J.H., Kim D.H., Lim Y.-H., Shin C.H., Lee Y.A., Kim B.-N., Kim J.I., Hong Y.-C. (2021). Childhood Obesity-Related Mechanisms: MicroRNome and Transcriptome Changes in a Nested Case-Control Study. Biomedicines.

[37] Bonilauri B., Camillo-Andrade A.C., Santos M.D.M., Fischer J.d.S.d.G., Carvalho P.C., Dallagiovanna B. (2021). Proteogenomic Analysis Reveals Proteins Involved in the First Step of Adipogenesis in Human Adipose-Derived Stem Cells. Stem Cells Int..

[38] Granata I., Nardelli C., D’Argenio V., Tramontano S., Compare D., Guarracino M.R., Nardone G., Pilone V., Sacchetti L. (2020). Duodenal Metatranscriptomics to Define Human and Microbial Functional Alterations Associated with Severe Obesity: A Pilot Study. Microorganisms.

[39] Huang T., Yu L., Pan H., Ma Z., Wu T., Zhang L., Liu K., Qi Q., Miao W., Song Z. (2021). Integrated Transcriptomic and Translatomic Inquiry of the Role of Betaine on Lipid Metabolic Dysregulation Induced by a High-Fat Diet. Front. Nutr..

[40] Wang Z., Zhu M., Wang M., Gao Y., Zhang C., Liu S., Qu S., Liu Z., Zhang C. (2021). Integrated Multiomic Analysis Reveals the High-Fat Diet Induced Activation of the MAPK Signaling and Inflammation Associated Metabolic Cascades via Histone Modification in Adipose Tissues. Front. Genet..

[41] Zhang C., Wang Y., Kimura T., Al-Mrabeh A.H. (2024). Editorial: The role of GPCRs in obesity. Front. Endocrinol..

[42] Griffin M.J. (2022). On the Immunometabolic Role of NF-κB in Adipocytes. Immunometabolism.

